# Weekday-weekend patterns of physical activity and screen time in parents and their pre-schoolers

**DOI:** 10.1186/s12889-016-3586-8

**Published:** 2016-08-30

**Authors:** Dagmar Sigmundová, Erik Sigmund, Petr Badura, Jana Vokáčová, Lucie Trhlíková, Jens Bucksch

**Affiliations:** 1 Faculty of Physical Culture, Palacký University, Tr. Miru 117, 77111 Olomouc, Czech Republic; 2 Department of Prevention and Health Promotion, Bielefeld University, School of Public Health, PO Box 100131, D-33501 Bielefeld, Germany

**Keywords:** Step count, Yamax pedometer, Mother, Father, Kindergarten, Weekdays, Weekends, Family

## Abstract

**Background:**

This study focuses on the comparison of weekday/weekend parent-child behavioural patterns (step count (SC) and screen time (ST)) and answers the question of whether achieving the recommendations for daily SC (10,000) in parents also helps their preschool children achieve the recommended daily SC (11,500).

**Methods:**

The participants (278 parents aged 30–45 and their 194 children aged 4–7) were randomly recruited from 10 Czech public kindergartens. The participants recorded SC (pedometer Yamax Digiwalker SW-200) and ST duration (proxy-report) for seven consecutive days (≥8 h/day) during September–October 2014 and April–May 2015. Differences between weekdays/weekends SC or ST were tested using a paired *t*-test. The odds of achieving the recommended daily SC for children were estimated using general logistic regression separately for weekdays and weekends.

**Results:**

Only the mothers were found to have a significantly lower SC at weekends than on weekdays. All of the participants showed significantly more ST at weekends than on weekdays (daughters: 78.6 vs. 45.7 min/day, *p* < 0.001; sons: 78.8 vs. 55.8 min/day; mothers: 93.0 vs. 68.3 min/day; and fathers: 116.6 vs. 87.5 min/day). Daughters and sons were significantly more likely to achieve daily SC recommendation if a) the SC on weekdays during the daily routine in kindergarten exceeded the median of kindergarten SC or b) at weekends if their mother (OR: 9.67, 95 % CI: 3.57–26.23) exceeded 10,000 steps a day.

**Conclusions:**

Especially at weekends, preschoolers have higher odds of meeting the recommended 11,500 steps per day when their mother reaches 10,000 steps per day and this is independent of the amount of parents’ ST. Moreover, physical activity in kindergarten helps preschool children meet the 11,500 recommended steps per day on weekdays. Therefore, interventions to promote physical activity in preschoolers should focus on kindergartens and encourage involvement of their families.

## Background

According to the Socialization Model of Children’s Behaviour [1], which is based on the Social Cognitive Theory [2], parents have a direct influence on their child’s behaviour and also on the body weight [3–6], physical activity (PA) [5, 7–9] and screen time (ST) of children [9–11]. The prominent questionnaire studies show the importance of parental support for the implementation of PA in their children [12–14]. Moreover an active lifestyle of parents is associated with a lower risk of overweight of their children [15, 16]. The pilot Czech study revealed existence of the positive relationship between PA of parents and their school-aged children. Reversely, higher sedentary behaviour of parents is linked to higher sedentary behaviour in children [17] with stronger significant association observed during weekends than on weekdays [18]. However, there is a lack of studies dealing with relationship between physical activity behaviour of preschool children and their parents.

The association of objectively measured PA among both parents (not only mothers [19, 20]) and their preschool children is also rarely examined [21, 22]. Moreover, it is not confirmed that the achievement of recommendations for daily PA in parents helps increase the chances of achieving daily PA recommendation in their preschoolers. This issue was recently highlighted within a consensus paper as one of the ten important research questions related to PA in preschool children [23]. More in-depth research is needed on the prevalence of meeting PA guidelines for preschool children and excessive sedentary behaviour, including the need to detect patterns and correlates of PA for preschool children in kindergarten, during their leisure time on weekdays and also at weekends [23].

Since 1990, the rise in number of overweight and obese preschoolers has been dramatic worldwide and currently poses serious health-related problems [24]. Therefore, it is desirable to identify effective interventions and programs to encourage energy-balanced behaviour to reverse this unfavourable trend toward overweight/obesity in preschool children [5, 9–11, 25–27]. Recommendations for designing PA-related interventions for preschoolers and interventions to reduce overweight/obesity call for involvement of both parents [25–27] in order to better integrate the children’s families into kindergarten-based interventions [28–31]. Additionally, preschool children whose parents consistently limited television viewing spent significantly less time in this behaviour and total ST [10, 11]. Recent systematic reviews on interventions aimed to reduce ST also recommended including a strong family component to improve the success of interventions [32, 33].

While in economically developed countries, including the countries of Western and Northern Europe, there are verified PA promotion programs and obesity-reducing interventions for preschool children [25, 26, 28–30, 34, 35], in the countries of Central and Eastern Europe, such programs/interventions are introduced much more rarely. Designing of PA promotion programs and obesity-reducing interventions, especially in preschool children, should be preceded by detecting patterns of energy-balanced behaviour of parents and their children [9–11] in different parts of the day, the week and the seasons [36]. Based on this knowledge, interventionist can develop programs that fit with the needs of pre-schoolers as a prerequisite of their long-term effects.

The aim of the present study is to reveal weekdays/weekends parents-child behaviour patterns (SC and ST). The second aim is to answer the question of whether achieving the recommendations for daily SC of parents also helps to achieve the recommendations for daily SC for their preschool children.

## Methods

### Participants

The parents of 4- to 7-year-old children were invited to enrol their children in the study after attending a detailed presentation on the design and purpose of the survey at a joint meeting with researchers, teachers and school administrators at each of the invited kindergartens. All families involved in the survey gave their written informed consent and participated in the study voluntarily and without any financial incentive. All participants who completed the survey received individual graphical feedback on their results. Ethical approval to conduct the study was obtained from the Ethics committee of the Faculty of Physical Culture, Palacký University, on 10^th^ December 2014 under reg. no. 57/2014.

Participants were recruited from ten public kindergartens in the Moravian region of the Czech Republic. The kindergartens involved in the present study were selected from the same settlement units as in the previous survey in 2005, thus covering all the administrative regions of Moravia and including kindergartens both form cities and rural areas [37]. Overall, more than 70 % of the total surveyed respondents agreed to participate in the research and more than 60 % of them completed monitoring of weekly PA, screen time and anthropometric testing (Table 1). For explanation of at least 10 % of variance in the dependent variable from independent variable, we determined minimal sample size per group (*n* > 70) with sample power of at least 80 % [38].

**Table 1:** Descriptive characteristics (number; percentages; means and standard deviations) by gender

	Parents		Children	
	Mothers	Fathers	Daughters	Sons
Number (%) of addressed respondents	234 (100 %)	181 (100 %)	141 (100 %)	155 (100 %)
Number (%) of participants who provided informed consent/were given pedometers	183 (78.21 %)	130 (71.82 %)	103 (73.05 %)	120 (77.42 %)
Number (%) of returned pedometers meeting validation criteria	166 (70.94 %)	112 (61.88 %)	88 (62.41 %)	106 (68.39 %)
Anthropometric variables of participants with valid pedometer data	(*n* = 166)	(*n* = 112)	(*n* = 88)	(*n* = 106)
Age (years)	36.06 ± 4.28	38.71 ± 4.89	5.58 ± 0.84	5.63 ± 0.86
Body height (cm)	167.65 ± 6.15	180.42 ± 6.83	116.28 ± 10.47	117.51 ± 7.64
Body weight (kg)	67.91 ± 11.66	84.88 ± 12.19	20.52 ± 3.99	21.37 ± 3.83
BMI (kg/m^2^)	24.18 ± 4.12	26.03 ± 3.26	15.22 ± 2.43	15.43 ± 1.81
Overweight^a,c^	25.61 %	52.73 %	11.36 %	8.49 %
Obesity^b,d^	10.98 %	10.00 %	9.09 %	9.43 %

*n* number of participants; *BMI* body mass index

^a^overweight or ^b^obesity in children represents a BMI from 85th to 97th or greater than 97th percentile of WHO growth charts [48, 49]; ^c^overweight or ^d^obesity in parents represents a BMI from 25 kg/m^2^ to 29.9 kg/m^2^ or greater than or equal to 30 kg/m^2^ [50]

All the kindergartens had similar indoor/outdoor kindergarten environment (separate houses surrounded by a grassy garden, with a children’s sandpit and an age-appropriate children’s playground) and the same minimum daily PA routine (a daily 30–60-min walk outdoors and 20 min of indoor exercise steps and dance variations, competitive and coordination movement games, relaxation or breathing activities, and other types of exercise) [39]. Measurements were carried out during spring (April/May) and autumn (September/October) of 2015.

### Measures

#### Pedometer

SC in children and parents were assessed with unsealed Yamax Digiwalker SW-200 pedometers (Yamax Inc., Tokyo, Japan), which is an unobtrusive tool, sized 1.9 × 3.9 × 5.2 cm, that uses a horizontal spring-suspended mechanical lever to measure vertical oscillations in movement. Validity and reliability of the SC of the Yamax Digiwalker SW-200 pedometer in preschool children has been previously evaluated by concurrent monitoring using Children’s Activity Rating Scale (CARS) [40–42]. The high validity of the Yamax Digiwalker SW-200 SC in adults in free-living conditions was confirmed by comparison with the ActivPAL during one working day (−240 SC per day, −5.9 % error deviation, ICC = 0.96, *p* < 0.01) and good test-retest reliability was confirmed on a 30-min treadmill walking test under laboratory conditions (ICC = 0.71, *p* < 0.01) [43]. The output variables that were analysed from the pedometer were overall daily SC and SC in kindergarten (occupational activities for parents). The daily SC is the value of the aggregate amount of steps shown on the display device of the pedometer-wearing participant during entire monitoring day, i.e. between the morning (pedometer mounted on) and evening (pedometer taken off). Kindergarten (employment) SC represents the amount of steps over time spent in kindergarten (occupational activities).

#### Logbook

The parent of each participating child involved was asked to record his/her own, as well as his/her child’s values of SC and duration of ST in a personalised family logbook. The personalised family logbook included one table for recording daily wear time and values of SC (morning after waking up, start and end of kindergarten for children (paid occupational activities for parents), and in evening before going to bed) and another table for recording the duration of sitting and lying while watching TV (DVD, video), as well as sitting and lying in front of a PC (notebook, tablet, smartphone) separately for all family members. The accuracy of recording the duration of ST was fixed at 10 min. Parents’ proxy-report of time of duration of TV watching of their 5–6-year-old children shows acceptable 7- to 14-day test-retest reliability (ICC = 0.78; 95 % CI, 0.69–0.84, *p* < 0.001) for detecting sedentary behaviour on usual weekdays and weekend days [44]. The output logbook-derived variable was the daily duration of ST (minutes). The variable of daily ST represented a sum of sitting and lying while watching TV (DVD, video) and sitting and lying in front of a PC (notebook, tablet, smartphone).

#### Anthropometric covariates

At a joint meeting with the researchers in kindergarten, the parents were asked to record their own, as well as child’s birthdate, gender, and body weight and height at home in a personalised family list before the start of monitoring of SC and ST. Parents were instructed how to measure body weight and height at home. Self-reported body weight and height can be used to estimate health risks associated with variations in BMI in adults [45]. In children this procedure is justified as home measurement of body weight and height of preschool children by their parents was verified by comparison with laboratory measurements (weight –ICC = 0.94, 95 % CI, 0.86–0.97; height, ICC = 0.95, 95 % CI, 0.94–0.96) in 297 Belgian pre-schoolers aged 3–7 years [46]. The chronological age of the participants was calculated from the date of birth until the first day of PA monitoring.

### Procedure

The week before the start of PA monitoring, participants were given the opportunity to familiarise themselves with the Yamax pedometers during a joint meeting at each of the participating kindergartens. The parents and teachers received written instructions for proper pedometer use, for recording in the family logbook, and for anthropometry measurement. They were instructed to ensure that children wore the pedometer on their right hip as long as possible during all waking hours except water activities and sleeping [37, 39].

During the morning of the first day of the 8-day PA monitoring period, the children in kindergarten received their pedometers. The pedometers were not sealed, but participants were instructed to open the pedometers only for the four SC recording times per weekday (two times per weekend – only morning and evening). The data for the first day were omitted because the recording of the first day was incomplete and to minimize the influence of possible behaviour change (e.g. effect novelty, reactivity). To be included in the analysis, the pedometer had to be worn for at least eight hours a day over at least four weekdays and two weekend days [47].

### Data analysis

Descriptive characteristics are presented in Table 1 as the means ± standard deviations (SD) or 95 % confidence intervals (95 % CI). Body mass index (BMI) (kg/m^2^) was calculated as body weight (kg) divided by the square of body height (m). Age-specific cut-off points, according to the World Health Organization [48, 49], were used to define the prevalence of children’s obesity. Overweight or obesity in children represents a BMI from the 85th to 97th or greater than the 97th percentile of the WHO growth charts [48, 49]. Overweight or obesity in parents represents a BMI from 25 kg/m^2^ to 29.9 kg/m^2^ or greater than or equal to 30 kg/m^2^ [50]. The chi-square test was used to assess gender-differences in overweight/obesity in preschoolers (daughters and sons), as well as their parents (mothers and fathers). Values of daily SC under 1000 or exceeding 30,000 were truncated to these recommended limit values, respectively [47, 51], and included in the analysis. Daily SC recommendation for preschool children represents a value of 11,500 steps/day [52] and for adults it is a value of 10,000 steps/day [53]. An excessive ST for children and adults was defined as two or more hours/day [54, 55]. Differences between weekdays/weekends SC or ST in parents and children, separately for gender, were tested using a paired *t*-test. Testing the difference in the percentage of participants achieving SC recommendations between weekdays and weekends were performed using the chi-square test. Logistic regression models (Enter method) were used to investigate whether parental achievement of the daily recommendation of SC and non-excessive ST on weekdays and weekend days (the two analyses separately) were associated with children’s achievement of daily SC. These analyses were adjusted for children’s and their parents’ age and overweight/obesity as categorical variables. First, we assessed the association of mothers’ SC and ST with their child’s SC on weekdays and weekend days. Then, we ran the analyses again for the father-child pairs. The data were analysed in total for all kindergartens because two-step cluster analysis found no indication for clustering by kindergarten. The Statistical Package for the Social Sciences (SPSS) for Windows v.22 software (IBM SPSS, Inc., Chicago, IL, USA) was used for data management and all statistical analyses. The significance level was set to *p* < 0.05 (2-tailed).

## Results

Prevalence of obesity was similarly about 10 % both in adult and child participants regardless of genders. However, we observed significantly higher percentage of parents reporting general excess weight, with almost one in four mothers and over half fathers being overweight. In children of both genders it was around 10 %.

**Fig. 1:**
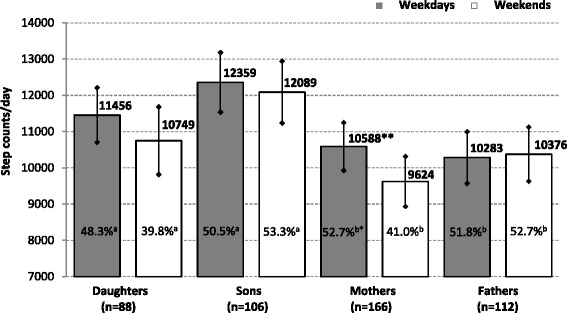
Comparison of parents’ and children’s pedometer-determined daily step counts (mean and 95 % confidence intervals) on weekdays and weekend days by gender. Legend: *n* number of participants; *%* ^*a*^ percentages of children who met step counts recommendation of 11,500 steps/day; *%* ^*b*^ percentages of parents who met step counts recommendation of 10,000 steps/day. Statistical significance of differences in daily step counts (percentages of meeting the step counts recommendations) between weekdays and weekends using paired *t*-test (chi-square test) is expressed as *p < 0.05 and **p < 0.01

The weekdays/weekends PA patterns, represented by SC, refer to overall slightly higher PA on weekdays than on weekends, except for fathers (Fig. 1). However, only mothers were found to reach significantly (*p* < 0.01) higher daily SC on weekdays than at weekends. Mothers were also the single study group with a significant difference in the percentage reaching SC recommendations of 10,000 steps per day (Fig. 1), thus being significantly less active at weekends. On weekdays no significant differences were found among the percentages of children and parents who met SC recommendations (11,500 steps per day for preschool children [52] and 10,000 steps per day for adults [53]). At weekends, a significantly (*p* < 0.05) lower percentage of females (mothers or daughters) than males (sons or fathers) reached the SC recommendations (Fig. 1). During the daily routine in kindergarten, daughters (sons) performed an average of 5455 (6221) SC per weekday, which accounted for 47.43 % (54.10 %) of the daily recommended 11,500 SC per day [43]. Similarly, during occupational activities on weekday mothers (fathers) reached 46.48 % (52.85 %) of daily recommended 10,000 SC per day [44], which represents, on average, 4921 (5285) SC per weekday.

**Fig. 2:**
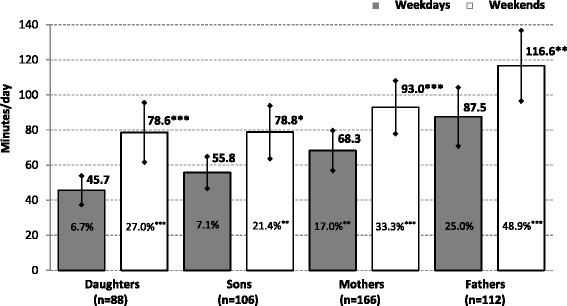
Comparison of parents’ and children’s logged daily screen time (mean and 95 % confidence intervals) on weekdays and weekend days separated by gender. Legend: *n* number of participants; *%* percentages of children/parents who exceeded 2 h a day of screen time. Statistical significance of differences in daily screen time (percentages of excessive screen time) between weekdays and weekends using a paired *t*-test (chi-square test) is expressed as *p < 0.05, **p < 0.01 and ***p < 0.001

Daily ST between weekday and weekends is depicted in Fig. 2. Regardless of gender, both the parents and children showed significantly showed significantly more ST on weekends than on weekdays. At weekends we also observed a significantly higher proportion of daughters, sons, mothers and fathers exceeding two or more hours of ST per day, compared with weekdays (Fig. 2). On weekdays, 6.7 % of preschool girls and 7.1 % of preschool boys spent more than 2 h a day in front of a television, video, computer or tablet/smart phone, while at weekends it was about four times as much in girls and three times as much in boys, respectively (Fig. 2).

Logistic regression analysis, adjusted for children’s and their parents’ age and overweight/obesity, revealed different odds of preschool children for meeting a daily SC recommendation (11,500 steps a day) separately for weekdays and weekends (Table 2). On weekdays, preschoolers are more than six times as likely to achieve the daily SC recommendation when the kindergarten daily routine accounted for more than the median SC (*p* < 0.001). At weekends, we found children to have almost ten times higher odds to meet the SC recommendation, when their mothers reached at least 10,000 steps a day (Table 2). The excessive ST (≥2 h per day) of parents does not affect the likelihood of achieving preschoolers’ SC recommendation either on weekdays or at weekends.

**Table 2:** Logistic regression analysis: Odds ratios and 95 % confidence intervals for meeting the step count recommendations in children on weekdays and weekend days, separately for mother-child and father-child pairs included in the model (controlled for children’s and their parents’ age and overweight/obesity)

	Step counts (SC) recommendation 11,500 steps/day											
	Weekdays						Weekends					
	%^a^	OR	95 % CI	%^a^	OR	95 % CI	%^a^	OR	95 % CI	%^a^	OR	95 % CI
	Mother in the model			Father in the model			Mother in the model			Father in the model		
Parent	*n* = 115			*n* = 74			*n* = 115			*n* = 74		
Step counts												
< 10,000 steps/day	51.9	Ref.		50.0	Ref.		31.9	Ref.		50.0	Ref.	
≥ 10,000 steps/day	60.6	1.26	0.52–3.10	71.8	2.63	0.83–8.38	77.6	9.67***	3.57–26.23	70.7	2.70	0.91–8.02
Screen time												
< 2 h per day	56.3	Ref.		59.6	Ref.		49.4	Ref.		57.9	Ref.	
≥ 2 h per day	59.1	1.36	0.43–4.31	65.0	2.18	0.45–10.62	53.8	0.64	0.22–1.82	64.1	1.83	0.55–6.04
Children	*n* = 115			*n* = 74			*n* = 115			*n* = 74		
Gender												
Boys	61.4	Ref.		61.9	Ref.		56.1	Ref.		69.0	Ref.	
Girls	52.5	0.69	0.28–1.67	60.0	0.75	0.21–2.62	45.9	0.60	0.25–1.47	51.4	0.33*	0.11–0.99
Daily screen time												
< median	65.5	Ref.		71.4	Ref.		54.0	Ref.		67.7	Ref.	
≥ median	48.3	0.37*	0.15–0.90	52.4	0.19*	0.43–0.85	47.5	1.19	0.47–3.03	56.5	0.32	0.09–9.33
Kindergarten												
Step counts												
< median	36.1	Ref.		38.9	Ref.		39.3	Ref.		50.0	Ref.	
≥ median	78.9	6.20***	2.6–14.9	80.5	8.78***	2.61–29.60	63.2	2.63*	1.09–6.34	70.7	3.05	0.99–6.66
Nagelkerke R^2^		0.30**			0.38**			0.35***			0.25	

*SC*, step counts; *ST*, screen time; *%* ^*a*^, proportion of children (daughter, sons) who met the pedometer-based recommendation for daily step counts 11,500 steps/day in a given row (e.g. 60.6 % of the children, whose mothers reached ≥ 10,000 steps/day on weekdays, met the recommendation of 11,500 steps/day); *OR*, odds ratio; *95 % CI*, confidence interval; *Ref.*, reference group

*p < 0.05; **p < 0.01; ***p < 0.001; *R* ^*2*^, Nagelkerke coefficient of determination, logistic model, Enter method

## Discussion

While previous research has examined the association between weekdays/weekends parent-child PA [56] or parent-child ST [14], relatively few studies to date have examined the association between weekdays/weekends parent-child PA and ST at once [18, 57]. Especially evidence on preschool children is scarce [5]. Thus far, no one has answered, to the best of our knowledge, the question of whether parents who achieve the recommendations for daily SC also help their preschool children achieve the recommendations for daily SC. The present study addressed this issue by the simultaneous monitoring of children’s and their parents’ PA using a pedometer and self-reported SC for at least 8 h a day during one school/occupational activities week involving both weekend days. Overall, we found that preschoolers have higher odds of meeting the recommended 11,500 steps per day when their mother reached 10,000 steps per day and this is independent of amount of parents’ ST. Moreover, PA in kindergarten also helps preschool children meet the 11,500 recommended steps per day on weekdays. These findings highlight the importance of family- and kindergarten-based intervention for promoting PA.

Regarding the assessment of participants’ PA patterns, in line with previous findings preschool boys were slightly more active than girls [21, 58], and children were slightly more active than parents [21, 51]. However, unlike previous pedometer-based studies [58, 59] in children and fathers there were no significant differences between weekdays and weekends PA. This finding in terms of pre-schoolers have been confirmed in other Czech pedometer-based studies [37, 60]. Possible reasons could be traced back to a tradition of a wide range of inexpensive leisure time PA-promoting programs and clubs for preschoolers; the availability of free age-adapted children’s playgrounds and parks; and the emerging private sector focusing on family health, wellness and recreation. All these issues together can effectively assist with increase of leisure-time and weekend PA of pre-schoolers [39]. However, this health-related positive pattern of comparable amount of PA in weekdays and weekends in pre-schoolers does not persist after entering primary school and years of compulsory schooling [37, 39, 57, 60]. This might indicate that parents spend more time with their children when they are younger and, thus, the possible influence on their children’s’ PA gets weaker with increasing age of the children.

ST unveiled a completely different weekdays/weekends pattern than for PA in all groups of participants, both in parents and their children. While on weekdays preschool children spend, on average, less than 1 h in front of a television, video, computer or tablet/smart phone, at weekends it is more than 78 min a day. In other words, on weekdays only 6.7 % of girls and 7.1 % of boys exceeded 2 h of ST, but on weekends 27.0 % of girls and 21.4 % of boys’ parents reported excessive ST (≥ 2 h/day). However, the prevalence of excessive daily ST for Czech preschoolers is still lower than the excessive daily ST of American [61, 62] and Canadian preschoolers [63]. Parents were also found to have considerable higher amounts of ST at weekends than on weekdays. Almost twice as many fathers had excessive ST at weekends, compared with weekdays. The same pattern of a significantly higher amount of weekend ST than on weekdays was observed also in 5- to 6-year-old English children and their parents [14], as well as in Czech families with children aged 9–12 years [57]. Intuitively, all members of families with preschool children spend more free time together and share more activities during weekend days than during weekdays. However, we are not able to differentiate between activities (SC or ST) which have been done together as a family or alone without a family context [57, 64].

The relationship between the behaviour (PA and ST) of parents and their preschool children has recently been assessed in the literature [4, 5, 8–10, 19, 21, 22] but there is a need to answer the question whether parents who achieve the recommendations for daily SC also help their preschool children achieve the recommendations for daily SC. Consistent with previous findings [4, 5, 19, 65], our study showed that parents have a significant impact on physical activity in their preschool children on weekdays and weekends. A previous study found that with one physically active parent (mother or father) the relative odds ratio of being active for his or her child was between 2.0 (mother) or 3.5 (father), in the case of both active parents the relative odds of being active for the children was found to be 5.8 [21]. There was no distinction between level of PA by gender of parents and children or between the type of day (weekday versus weekend day) [21]. The results of our study support the assumption that if mothers meet recommendations for daily step counts of 10,000 steps/day [53], then their children are notably more likely to meet the PA recommendation of 11,500 steps/day too [52], regardless of children’s gender and the amount of parents’ ST. However, this was observed to be significant only on weekend days, points to the importance of promoting PA in families with a special focus on weekends.

The interesting and new finding observed in this study was that PA during kindergarten markedly assists boys and girls in achieving the recommended 11,500 steps/day [52] on weekdays and partly also at weekends. In light of the general low levels of PA in children, it is not surprising that increased PA in kindergarten significantly contributes to overall higher PA on weekdays. However, our finding highlighted the necessity of kindergartens to offer as many opportunities as possible for structured and unstructured physical activities to enable a substantial amount of SC per day. Introduction of PA-promoting curricula in kindergartens, thus, might be an efficient way to encourage PA in a large number of preschoolers and kindergartens should be aware of their PA-promoting role.

Our study also showed that, in general, amount of ST in parents or children was not associated with meeting SC recommendations. Therefore, our study did not support a displacement hypothesis of PA through ST, which is in line with a lot of other studies [66]. In this sense we could confirm the assumption that it is important to develop interventions aimed to promote PA and to reduce ST as separate intervention targets [33]. This becomes even more important since both behaviours are independently associated with health outcomes in school-aged children [67]. Explaining this finding is not easy and further research is warranted on this issue.

### Strengths and limitations

The main strength of this study is the involvement of all family members in monitoring of the week-long ambulatory PA and the self-reported screen time. Strict criteria for inclusion in the data analysis (data only from children and their parents whose PA and screen time was monitored continuously for at least 8 h a day, on at least four weekdays and both weekend days) can be considered as the strength of the study. Another strength of the study is that the total amount of daily PA on weekdays is supplemented with information on the amount of PA in kindergarten (children) and occupational activities (parents).

The interpretation of the results and the formulation of conclusions of the study has to take existing limitations of the study into account. First, our study did not collect data on the socioeconomic status of families (including the educational level of parents), incomplete participating families (divorced parents, separate housing), and type of residence (apartment vs. family house) [3] and also did not take the number of children in the family and ages of participating children’s siblings into account. These factors might have indirectly influenced parents-child behavioural patterns, but they have not been investigated because of the disproportionate burden of parents regarding continuous monitoring of their own and their child’s weekly PA and screen time. Second, using a pedometer is a further limitation of the study. Although pedometers are recommended as a cost-effective, valid and reliable method that provides summary output of daylong ambulatory PA of preschool children [59, 68, 69], they are unable to accurately determine the type and intensity of PA [70]. However, pedometers may be used as a viable alternative to an accelerometer to estimate the moderate-to-vigorous PA [59, 69, 71] that relates to a health criterion and recommendations [68]. Third, the study presented only the total daily ST and did not analyse the structure and types of ST, especially for parents. The total amount of parents’ ST may depend on the type and daily period of occupational activities and the socioeconomic status of families.

Despite the aforementioned limitations, the results of this study extend the knowledge of parent-child PA behavioural patterns in families with preschool child and can assist in designing PA-promoting programs and weight-related interventions for young children.

## Conclusion

The results of this study provide evidence that advocates for the importance of promoting age-appropriate kindergarten-based PA-promoting programs with emphasis on involvement of families as a means of shaping positive attitude towards PA behaviour in preschool children. The study identified the following essential elements of an effective promotion of PA for pre-schoolers: a combination of PA program in kindergartens on weekdays and active PA behaviour in parents during the weekends (≥10,000 step per day) could help preschool children meet the recommended levels of daily PA (≥11,500 step per day) throughout the week. Future research is needed to assess the moderating effect of family-related variables (socioeconomic status, incompleteness of families, type of residence, and number, age, and PA level of siblings) in meeting PA health-related recommendations more thoroughly in preschoolers.

## Abbreviations

BMI: Body mass index
CARS: Children’s Activity Rating Scale
CI: Confidence interval
ICC: Intra class correlation
OR: Odds ratio
PA: Physical activity
Ref.: Reference group
SC: Step counts
ST: Screen time

## References

[1] Taylor WC, Baranowski T, Sallis JF, Dishman RK (1994). Family determinants of childhood physical activity: a social-cognitive model. Advances in exercise adherence.

[2] Bandura A (1986). Social foundations of thought and action: a social cognitive theory.

[3] Parikka S, Mäki P, Levälahti E, Lehtinen-Jacks S, Martelin T, Laatikainen T (2015). Associations between parental BMI, socioeconomic factors, family structure and overweight in Finnish children: a path model approach. BMC Public Health.

[4] Sijtsma A, Sauer PJ, Corpeleijn E (2015). Parental correlations of physical activity and body mass index in young children- the GECKO Drenthe cohort. Int J Behav Nutr Phys Act.

[5] Vollmer RL, Adamsons K, Gorin A, Foster JS, Mobley AR (2015). Investigating the relationship of body mass index, diet quality, and physical activity level between fathers and their preschool-aged children. J Acad Nutr Diet.

[6] Lin Y-C, Wu JC-L, Chiou S-T, Chiang T-L (2015). Healthy living practices in families and child health in Taiwan. Int J Public Health.

[7] Alderman BL, Benham-Deal TB, Jenkins JM (2010). Change in parental influence on children’s physical activity over time. J Phys Act Health.

[8] Yao CA, Rhodes RE (2015). Parental correlates in child and adolescent physical activity: a meta-analysis. Int J Behav Nutr Phys Act.

[9] Carson V, Stearns J, Janssen I (2015). The relationship between parental physical activity and screen time behaviors and the behaviors of their young children. Pediatr Exerc Sci.

[10] Downing KL, Hinkley T, Hesketh KD (2015). Associations of parental rules and socioeconomic position with preschool children’s sedentary behaviour and screen time. J Phys Act Health.

[11] Hnatiuk JA, Salmon J, Campbell KJ, Ridgers ND, Hesketh KD (2015). Tracking of maternal self-efficacy for limiting young children’s television viewing and associations with children’s television viewing time: a longitudinal analysis over 15-months. BMC Public Health.

[12] Jago R, Davison KK, Thompson JL, Page AS, Brockman R, Fox KR (2011). Parental sedentary restriction, maternal parenting style, and television viewing among 10- to 11-year-olds. Pediatrics.

[13] Trost SG, Sallis JF, Pate RR, Freedson PS, Taylor WC, Dowda M (2003). Evaluating a model of parental influence on youth physical activity. Am J Prev Med.

[14] Jago R, Thompson J, Sebire S, Wood L, Pool L, Zahra J, Lawlor D (2014). Cross-sectional associations between the screen-time of parents and young children: differences by parent and child gender and day of the week. Int J Behav Nutr Phys Act.

[15] Erkelenz N, Kobel S, Kettner S, Drenowatz C, Steinacker JM, The Research Group “Join the Healthy Boat – Primary S (2014). Parental activity as influence on children‘s BMI percentiles and physical activity. J Sports Sci Med.

[16] Thibault H, Contrand B, Saubusse E, Baine M, Maurice-Tison S (2010). Risk factors for overweight and obesity in French adolescents: Physical activity, sedentary behavior and parental characteristics. Nutrition.

[17] Sigmund E, Turoňová K, Sigmundová D, Přidalová M (2008). The effect of parent’s physical activity and inactivity on their children’s physical activity and sitting. Acta Univ Palacki Olomuc Gymnica.

[18] Sigmundová D, Sigmund E, Vokáčová J, Kopčáková J (2014). Parent-child associations in pedometer-determined physical activity and sedentary behaviour on weekdays and weekends in random samples of families in the Czech Republic. Int J Environ Res Public Health.

[19] Hesketh KR, Goodfellow L, Ekelund U, McMinn AM, Godfrey KM, Inskip HM, Cooper C, Harvey NC, van Sluijs EM (2014). Activity levels in mothers and their preschool children. Pediatrics.

[20] Maltby AM (2015). Exploring mothers’ influence on preschoolers’ physical activity levels and sedentary time.

[21] Moore LL, Lombardi DA, White MJ, Campbell JL, Oliveria SA, Ellison RC (1991). Influence of parents’ physical activity levels on activity levels of young children. J Pediatr.

[22] Ruiz R, Gesell SB, Buchowski MS, Lambert W, Barkin SL (2011). The relationship between Hispanic parents and their preschool-aged children’s physical activity. Pediatrics.

[23] Pate RR, O’Neill JR, Brown WH, McIver KL, Howie EK, Dowda M (2013). Top 10 research questions related to physical activity in preschool children. Res Q Exerc Sport.

[24] de Onis M, Blössner M, Borghi E (2010). Global prevalence and trends of overweight and obesity among preschool children. Am J Clin Nutr.

[25] Ling J, Robbins LB, Wen F, Peng W (2015). Interventions to increase physical activity in children aged 2-5 years: a systematic review. Pediatr Exerc Sci.

[26] Ling J, Robbins LB, Wen F (2016). Interventions to prevent and manage overweight or obesity in preschool children: a systematic review. Int J Nurs Stud.

[27] Schoeppe S, Trost SG (2015). Maternal and paternal support for physical activity and healthy eating in preschool children: a cross-sectional study. BMC Public Health.

[28] Puder JJ, Marques-Vidal P, Schindler C, Zahner L, Niederer I, Bürgi F, Ebenegger V, Nydegger A, Kriemler S (2011). Effect of multidimensional lifestyle intervention on fitness and adiposity in predominantly migrant preschool children (Ballabeina): cluster randomised controlled trial. BMJ.

[29] Roth K, Kriemler S, Lehmacher W, Ruf KC, Graf C, Hebestreit H (2015). Effects of a physical activity intervention in preschool children. Med Sci Sports Exerc.

[30] Fitzgibbon ML, Stolley MR, Schiffer L, Kong A, Braunschweig CL, Gomez-Perez SL, Odoms-Young A, Van Horn L, Christoffel KK, Dyer AR (2013). Family-based hip-hop to health: outcome results. Obesity.

[31] Morris H, Skouteris H, Edwards S, Rutherford L (2015). Obesity prevention interventions in early childhood education and care settings with parental involvement: a systematic review. Early Child Dev Care.

[32] Marsh S, Foley LS, Wilks DC, Maddison R (2014). Family-based interventions for reducing sedentary time in youth: a systematic review of randomized controlled trials. Obes Rev.

[33] Biddle SJH, Petrolini I, Pearson N (2014). Interventions designed to reduce sedentary behaviours in young people: a review of reviews. Br J Sports Med.

[34] Bellows LL, Davies PL, Anderson J, Kennedy C (2013). Effectiveness of a physical activity intervention for head start preschoolers: a randomized intervention study. Am J Occup Ther.

[35] Nemet D, Geva D, Eliakim A (2011). Health promotion intervention in low socioeconomic kindergarten children. J Pediatr.

[36] Laukkanen A, Pesola AJ, Heikkinen R, Sääkslahti AK, Finni T (2015). Family-based cluster randomized controlled trial enhancing physical activity and motor competence in 4-7-year-old children. PLoS ONE.

[37] Sigmund E, Sigmundová D, El Ansari W (2009). Changes in physical activity in pre-schoolers and first-grade children: longitudinal study in the Czech Republic. Child Care Health Dev.

[38] Hopkins WG (2006). Estimating sample size for magnitude-based inferences. Sportscience.

[39] Sigmund E, Sigmundová D (2014). School-related physical activity, lifestyle and obesity in children.

[40] McKee DP, Boreham CA, Murphy MH, Nevill AM (2005). Validation of the Digiwalker™ pedometer for measuring physical activity in young children. Pediatr Exerc Sci.

[41] Louie L, Chan L (2003). The use of pedometry to evaluate the physical activity levels among preschool children in Hong Kong. Early Child Development and Care.

[42] Oliver M, Schofield GM, Kolt GS, Schluter PJ (2007). Pedometer accuracy in physical activity assessment of preschool children. J Sci Med Sport.

[43] Kooiman TJM, Dontje ML, Sprenger SR, Krijnen WP, van der Schans CP, de Groot M (2015). Reliability and validity of ten consumer activity trackers. BMC Sports Sci Med Rehab.

[44] Salmon J, Campbell KJ, Crawford DA (2006). Television viewing habits associated with obesity risk factors: a survey of Melbourne schoolchildren. Med J Aust.

[45] Strommel M, Schoenborn C (2009). Accuracy and usefulness of BMI measures based on selfreported weight and height: findings from the NHANES & NHIS 2001-2006. BMC Public Health.

[46] Huybrechts I, Himes JH, Ottevaere C, De Vriendt T, De Keyzer W, Cox B, Van Trimpont I, De Bacquer D, De Henauw S (2011). Validity of parent-reported weight and height of preschool children measured at home or estimated without home measurement: a validation study. BMC Pediatr.

[47] Rowe DA, Mahar MT, Raedeke TD, Lore J (2004). Measuring physical activity in children with pedometers: Reliability, reactivity, and replacement of missing data. Pediatr Exerc Sci.

[48] Growth reference data for 5-19 years. WHO reference 2007. [http://www.who.int/growthref/en/]. Accessed 21 Jan 2016.

[49] de Onis M, WHO Multicentre Growth Reference Study Group (2006). WHO child growth standards based on length/height, weight and age. Acta Paediatr.

[50] Obesity and overweight. Fact sheet No 311. [http://www.who.int/mediacentre/factsheets/fs311/en/]. Accessed 21 Jan 2016.

[51] Craig CL, Cameron C, Tudor-Locke C (2013). Relationship between parent and child pedometer-determined physical activity: a sub-study of the CANPLAY surveillance study. Int J Behav Nutr Phys Act.

[52] De Craemer M, De Decker E, De Bourdeaudhuij I, Verloigne M, Manios Y, Cardon G (2015). The translation of preschoolers’ physical activity guidelines into a daily step count target. J Sports Sci.

[53] Tudor-Locke C, Craig C, Brown W, Clemes S, De Cocker K, Giles-Corti B, Hatano Y, Inoue S, Matsudo S, Mutrie N (2011). How many steps/day are enough? For adults. Int J Behav Nutr Phys Act.

[54] Tremblay MS, Colley RC, Saunders TJ, Healy GN, Owen N (2010). Physiological and health implications of a sedentary lifestyle. Appl Physiol Nutr Metab.

[55] American Academy of Pediatrics, Committee on Public Education (2001). Children, adolescents, and television. Pediatrics.

[56] Jacobi D, Caille A, Borys J-M, Lommez A, Couet C, Charles M-A, Oppert J-M, Group FS (2011). Parent-offspring correlations in pedometer-assessed physical activity. PLoS ONE.

[57] Sigmund E, Sigmundová D, Baďura P, Voráčová J (2015). Relationship between Czech parent and child pedometer-assessed weekday and weekend physical activity and screen time. Cent Eur J Public Health.

[58] Brasholt M, Chawes B, Kreiner-Moller E, Vahlkvist S, Sinding M, Bisgaard H (2013). Objective assessment of levels and patterns of physical activity in preschool children. Pediatr Res.

[59] Cardon G, De Bourdeaudhuij I (2007). Comparison of pedometer and accelerometer measures of physical activity in preschool children. Pediatr Exerc Sci.

[60] Sigmund E, Croix MDS, Miklánková L, Frömel K (2007). Physical activity patterns of kindergarten children in comparison to teenagers and young adults. Eur J Pub Health.

[61] Tandon PS, Zhou C, Lozano P, Christakis DA (2011). Preschoolers’ total daily screen time at home and by type of child care. J Pediatr.

[62] Lampard AM, Jurkowski JM, Davison KK (2013). The family context of low-income parents who restrict child screen time. Childhood Obesity.

[63] Carson V, Tremblay MS, Spence JC, Timmons BW, Janssen I (2013). The Canadian sedentary behaviour guidelines for the early years (zero to four years of age) and screen time among children from Kingston, Ontario. Paediatrics and Child Health.

[64] Sisson SB, Church TS, Martin CK, Tudor-Locke C, Smith SR, Bouchard C, Earnest CP, Rankinen T, Newton RL, Katzmarzyk PT (2009). Profiles of sedentary behavior in children and adolescents: The US National Health and Nutrition Examination Survey, 2001–2006. Int J Pediatr Obes.

[65] Oliver M, Schofield GM, Schluter PJ (2010). Parent influences on preschoolers’ objectively assessed physical activity. J Sci Med Sport.

[66] Mansoubi M, Pearson N, Biddle SJH, Clemes S (2014). The relationship between sedentary behaviour and physical activity in adults: a systematic review. Prev Med.

[67] Rezende LFM, Rodrigues Lopes M, Rey-López JP, Matsudo VKR, Luiz OC (2014). Sedentary behavior and health outcomes: an overview of systematic reviews. PLoS ONE.

[68] Pagels P, Boldemann C, Raustorp A (2011). Comparison of pedometer and accelerometer measures of physical activity during preschool time on 3- to 5-year-old children. Acta Paediatr.

[69] Peters BP, Kate A, Abbey BM (2013). Validation of Omron™ pedometers using MTI accelerometers for use with children. Int J Exerc Sci.

[70] Rowlands AV, Eston RG (2007). The measurement and interpretation of children’s physical activity. Journal of Sports Science and Medicine.

[71] Hands B, Larkin D (2006). Physical activity measurement methods for young children: a comparative study. Meas Phys Educ Exerc Sci.

